# Handling deviating control values in concentration-response curves

**DOI:** 10.1007/s00204-020-02913-0

**Published:** 2020-09-23

**Authors:** Franziska Kappenberg, Tim Brecklinghaus, Wiebke Albrecht, Jonathan Blum, Carola van der Wurp, Marcel Leist, Jan G. Hengstler, Jörg Rahnenführer

**Affiliations:** 1 Department of Statistics, TU Dortmund University, 44221 Dortmund, Germany; 2 Leibniz Research Centre for Working Environment and Human Factors (IfADo), TU Dortmund University, 44139 Dortmund, Germany; 3 Department of Biology, University of Konstanz, 78457 Constance, Germany

**Keywords:** Concentration-response curve, Dose-response curve, Viability assay, Deviating controls, 4pLL model, Simulation study

## Abstract

In cell biology, pharmacology and toxicology dose-response and concentration-response curves are frequently fitted to data with statistical methods. Such fits are used to derive quantitative measures (e.g. EC$_{20}$ values) describing the relationship between the concentration of a compound or the strength of an intervention applied to cells and its effect on viability or function of these cells. Often, a reference, called negative control (or solvent control), is used to normalize the data. The negative control data sometimes deviate from the values measured for low (ineffective) test compound concentrations. In such cases, normalization of the data with respect to control values leads to biased estimates of the parameters of the concentration-response curve. Low quality estimates of effective concentrations can be the consequence. In a literature study, we found that this problem occurs in a large percentage of toxicological publications. We propose different strategies to tackle the problem, including complete omission of the controls. Data from a controlled simulation study indicate the best-suited problem solution for different data structure scenarios. This was further exemplified by a real concentration-response study. We provide the following recommendations how to handle deviating controls: (1) The log-logistic 4pLL model is a good default option. (2) When there are at least two concentrations in the no-effect range, low variances of the replicate measurements, and deviating controls, control values should be omitted before fitting the model. (3) When data are missing in the no-effect range, the Brain-Cousens model sometimes leads to better results than the default model.

**Electronic supplementary material:**

The online version of this article (10.1007/s00204-020-02913-0) contains supplementary material, which is available to authorized users.

## Introduction

Concentration-response curves are often used to graphically describe the relationship between the concentration of a compound applied to cells and the resulting response. More general, any response of a cell or an organism to an exposure or stimulus can be modeled as a function of exposure time or as a function of a concentration or dose. Many types of assays in cell biology, pharmacology and toxicology generate such data. As reference, typically concentration 0 or exposure time $t_0=0$, respectively, is used. This reference is often called negative control, or simply control.

Fitting dose-response curves or concentration-response curves serves different purposes. Two main objectives are the determination of a benchmark dose or an effective concentration at which the curve drops by a certain percentage or to a specified absolute value, and the comparison of curves from different exposures. Both goals require suitable normalization of the curves, in practice often depending on the observed values for the control.

However, examination of the published toxicological literature shows many examples, where the values corresponding to the controls and the upper asymptote of concentration-response curves do not fit together (Krebs et al. 2018). This means that the response value for the control is different from the asymptotic response value for low concentrations. This situation is further called deviating controls. Note that deviating controls can only be undoubtedly identified, when an upper asymptote of a curve can be estimated with reasonable certainty, i.e. where a sufficient number of data points at very low (no effect) concentrations are available.

Real-life data can have several additional problems, the most frequent is that the upper asymptote is not defined at all by data in the range of the asymptote value, but it is rather derived by an extrapolation of data in the descending part of the curve. This situation can result from flawed experimental design, and it could be remedied by inclusion of more data points in the low-concentration range.

In the case of sufficient data points at very low concentrations, the explanation for deviating controls is less straightforward, as the controls do not fit to the curves despite an adequate experimental design (spacing of data points on the concentration axis). The most likely reasons are:

- Random variations of data points due to experimental imperfections
- Errors during the performance of the experiments (e.g. in producing stock dilutions)
- Variation in the concentration of solvents between samples
- Systematic deviation of endpoint readouts according to their position on culture plates or in analytical devices
- Systematic deviations due to the timing of sample preparation (e.g. incubation of cells, storage of solutions or during analysis, etc.)

Deviating controls raise the question how to appropriately deal with this situation. On the one hand, if not enough measurements for very low concentrations are available, controls are required to fit the asymptote of the concentration-response curve, and it is difficult to judge from observed data if the deviation is critical. On the other hand, the use of deviating controls can cause an extreme bias both for estimating the curves (or its parameters) and for estimating parameters derived from these curves, such as benchmark concentrations or EC values.

Different definitions of benchmark concentrations and EC values are used in the literature. Here, we use the term EC value in the following sense: $\text {EC}_{20}$ is the concentration, where an absolute viability of 80% is reached.

**Fig. 1:**
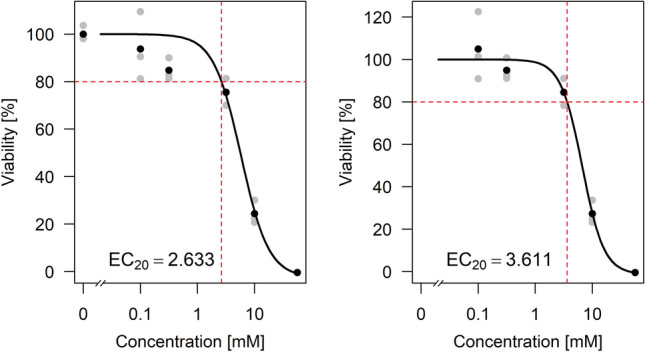
Concentration-response curves fitted to a part of the VPA dataset, normalizing to controls (left) and normalizing to the upper asymptote of the model fit and omitting controls (right). The fitted models are the methods ”3pLL” (left) and ”NoCtrl” (right) as explained in section Handling deviating controls. In the left plot, the fitted upper asymptote is higher relative to the data points and the fit results in a smaller EC$_{20}$ value (EC$_{20}=2.633$) compared to the right plot with EC$_{20}=3.611$

As an illustrating example we fitted two different concentration-response curves to the same dataset (Fig. 1). The dataset is part of the real dataset analyzed in detail in the Results section. Cells are treated with valproic acid (VPA), with control values and increasing concentrations. Here, for a subset with five concentrations (plus control) and three replicates, two non-linear sigmoidal curves are fitted. Apparently, the control values are higher than the response values for very low concentrations. In one case the data are normalized to the controls such that the mean control value is 100 and the upper asymptote of the curve is set to 100 (Fig. 1, left), and in the other case the control values are omitted and the data are normalized such that the upper asymptote corresponds to a value of 100 (Fig. 1, right).

The results show that the fitting procedure has a strong influence on the resulting estimate of the EC$_{20}$ value. When including the control values, the estimated value is 2.633, and when omitting them, the value is 3.611. By visual inspection, for this specific case, the right fit seems to be more appropriate. Guidance for a general procedure to identify the best fit is required.

In this paper, we analyze the extent of deviating controls in published concentration-response curves, and we present a comprehensive simulation study that analyzes the impact of deviating controls on the quality of the estimation of EC values. First, we present the literature study based on the journals ”Archives of Toxicology”, ”Toxicological Sciences”, and ”Toxicology in vitro” to determine the frequency and the extent of deviating controls in published toxicological assays.

Then we present four different strategies to handle deviating controls, including different modelling and normalization approaches and one approach that completely omits the controls. Many different modelling approaches have been proposed. Here, we focus on sigmoidal relationships between the concentration and the response.

We perform a simulation study with three different concentration regimes, and with different degrees of deviation of the controls and of variance of the replicate measurements. This leads to suggestions in which situations which strategy is most appropriate. Finally, we apply the methods to the raw data of a real data study with a large number of different concentrations to illustrate the insights from the simulation study.

## Materials and methods

### Literature review

We conducted a literature review in three leading international toxicological journals, ”Archives of Toxicology” (all issues from 2016–2018), ”Toxicological Sciences” (all issues from 2017–2018), and ”Toxicology in Vitro” (all issues from 2015–2017), to answer the following two questions:

- How often does the problem of deviating control values occur?
- How strong are the deviations in these cases?

We restricted our research to viability assays, where viability is defined in a broad sense, such that not only life and death is considered, but also activity (mostly mitochondrial activity), motility, contraction, or mitotic activity.

For such assays, we define a set of necessary criteria for including the curves in our analysis set:

- A concentration-response model is fitted to the data.
- Measurements for some (negative) controls are available.
- Measurements for at least four concentrations are available, in addition to the control.
- When neglecting the controls, the response values are monotonously decreasing with increasing concentration.
- For at least two of the concentrations (other than control), no effect can be observed, i.e. the difference between the corresponding response values is smaller than $10\%$ of the response value for the lowest concentration.
- For every concentration, at least three replicate values are available (technical or biological).

For the curves that fulfill these criteria, we looked up (if available in the corresponding article) or otherwise estimated both the average values of the controls and the value of an upper asymptote when omitting the controls. If a curve was fitted without the controls, the value of the upper asymptote was directly obtained from the corresponding figure or data, if available. Otherwise, the asymptote was estimated manually as an average of all response values in the no-effect range. The deviation of the controls was calculated in percent, based on a value of 100 for the asymptote. If Control represents the average of all individual control values and Fit represents the value of the upper asymptote for very small concentrations when omitting the controls, then the deviation is $\varDelta = (\mathtt{Control} - \mathtt{Fit})/\mathtt{Fit} * 100$.

Additional information was collected for each curve. For each concentration, the corresponding response value and the standard deviation of the replicates were determined, by estimating the values from the plotted data. If a standard deviation was smaller than 1 (for response values measured in percent values), then the value was set to 1. The average standard deviation for an entire curve was calculated as the median of the standard deviations for all concentrations except the control and was denoted by $\hat{\sigma }_\mathrm{med}$.

### Fitting concentration-response models

A parametric model is a statistical model with a finite number of parameters that have to be estimated from the data. We consider the situation, that for a set of concentrations including a negative control, measurements of several replicates (biological or technical) are available. Further, mostly, we assume that the lowest concentrations were chosen in a range, where no change in viability is expected to occur. We call these concentrations no-effect concentrations.

When fitting parametric concentration-response curves to data, after an initial background correction, often the first modelling step is to normalize the data with respect to the controls to obtain percentages. Then a curve can be fitted to the data, and a benchmark concentration, at which a specific relative response is observed, can be estimated. Here, we concentrate on effective concentrations with $\text {EC}_{\lambda }$ being the concentration where $(100 - \lambda )\%$ viability is attained.

For deviating controls, where the response values of the controls deviate from the response values of the no-effect concentrations, the upper asymptote of the curve fitted to all data including controls does not correspond to an effect of $100\%$ and therefore the EC values cannot be properly interpreted. For example, consider the $\text {EC}_{10}$ value, which corresponds to the intersection of the fitted curve with the value $90\%$. In the extreme case that the upper asymptote attains a value lower than $90\%$, the calculation of the $\text {EC}_{10}$ value is even impossible.

We propose four methods to deal with this situation. All methods are based on the assumption that the relationship between concentration and viability can be described by a function of the family of the log-logistic functions, which have a typical sigmoidal shape.

As a basic model for describing the relationship between concentration and response, we use the four-parameter log-logistic model (4pLL) (e.g. Ritz 2010). For a concentration *x*, $x \ge 0$, and four parameters *b*, *c*, *d*, *e*, with $e>0$, the model is defined by the relationship

$$\begin{aligned} f(x|b, c, d, e) = c + \frac{d - c}{1 + \exp (b(\log (x)-\log (e))}. \end{aligned}$$

The parameters *c* and *d* are the lower and the upper asymptote of the function, respectively, *b* is the slope parameter, and *e* is the inflection point. The parameter *e* also corresponds to the concentration where the half-maximal effect can be observed, i.e. a response of $\frac{c+d}{2}$. Often, especially for small data sets, the re-parameterisation $\tilde{e} = \log (e)$ is used. Note that the parameter *e* only coincides with our definition of the $\text {EC}_{50}$ value if $d=100$, $c=0$ and $b>0$, or if $d=0$, $c=100$ and $b<0$.

Fixing the parameter *d* to a value of 100 and restricting to $b>0$ yields a three-parameter log-logistic model (3pLL) with a prespecified upper asymptote of 100. Adding a fifth parameter $f \ge 0$ and extending the model to

$$\begin{aligned} f(x|b, c, d, e, f) = c + \frac{d - c + f \cdot x}{1 + \exp (b(\log (x)-\log (e))} \end{aligned}$$

yields the Brain-Cousens function (BC) which is no longer point-symmetric but can model a so-called hormesis effect, where higher response values than for the controls are observed in the low-concentration range (Brain and Cousens 1989). The size of the hormesis effect is determined by the value of the parameter *f*. The larger *f* the larger is the hormesis effect. In the BC model, the parameters *c* and *d* still represent the lower and the upper asymptote, respectively, but the parameters *b* and *e* do not have a direct interpretation as for the 4PLL model.

The curves were fitted numerically under the assumption of normally distributed response values by minimizing the sum of squared errors between the data points and the fitted function. We fitted the curves using the statistical software R, version 3.5.1 (R Development Core Team 2018), and the R-package drc (Ritz et al. 2015)

For the 3pLL and the 4pLL function we estimated the EC values based on the inverse of the model function. This means that for a fixed value of $\lambda$ the $\text {EC}_{\lambda }$ value is the concentration $\tilde{x}$ at which $f(\tilde{x}|b,c,d,\tilde{e}) = 100-\lambda$, that is

$$\begin{aligned} \tilde{x} = \exp (\tilde{e})\left( \frac{d-(100-\lambda )}{(100-\lambda )-c} \right) ^{\frac{1}{b}}. \end{aligned}$$

For the 3pLL function, in this formula *d* was replaced by the constant 100. For the BC model, EC values cannot be calculated with an inverse function. Instead, they are obtained by numerical optimization.

### Handling deviating controls

Based on the dose-response models from the family of the log-logistic functions, we propose four methods for fitting a model to the data with an upper asymptote of 100%, or respectively a maximum value of 100% for a present hormesis effect. The procedures and their acronyms used in this paper are:

- **4pLL**
  - Fit a 4pLL model to the original data
  - Normalize the data with respect to the upper asymptote
  - Fit a 4pLL model to the normalized data
- **3pLL**
  - Normalize the data with respect to the controls
  - Fit a 3pLL model with fixed value $d=100$ (for upper asymptote)
- **No Ctrl**
  - Fit a 4pLL model to the original data without the controls
  - Normalize the data with respect to the upper asymptote
  - Fit a 4pLL model to the normalized data without the controls
- **BC**
  - Fit a Brain-Cousens model to the original data
  - Normalize the data with respect to the upper asymptote or the maximal value of the fitted curve (maximal value is set to 100, if a hormesis effect is observed for the fit)
  - Fit a Brain-Cousens model to the normalized data

The methods ”4pLL” and ”3pLL” make full use of the control values with the difference that once the value of the upper asymptote and once the mean control value is used for normalizing the data. ”No Ctrl” completely ignores the control values, and ”BC” allows a more flexible fit for modelling curves in cases where a hormesis effect is observed at low concentrations before viability decreases at higher concentrations.

The second model fit for the methods ”4pLL”, ”No Ctrl” and ”BC” is only required since starting values for the optimization may be range-dependent and thus can lead to slightly different results. For the first three methods, the final upper asymptote corresponds to a viability of $100\%$. For the method ”BC”, if a hormesis effect is observed, the data is normalized with respect to the maximal value of the fitted curve.

In the choice of models considered, we concentrate on functions from the family of log-logistic functions as these are well-established in the field of toxicology for modelling concentration-response relationships where the response is viability of a cell. The methods ”3pLL” and ”No Ctrl” correspond to two intuitive ideas one might have when dealing with the problem of deviating controls. When using the ”3pLL” model, the upper asymptote is only determined by the control values, while for ”No Ctrl”, the controls are not considered at all for determining the value of the upper asymptote. In the ”4pLL” model, the importance of the controls lies between these two extremes, therefore these three methods cover a wide range of the weighting with which the controls should be included into the analysis. The ”BC” model includes the additional idea that in the case of negatively deviating controls, these deviations should not be compensated by some renormalization or by omission of the controls, but instead should be taken into account in the model.

### Design of the simulation study

In a simulation study we compare the four methods introduced above, considering several scenarios (subsequently called situations) with deviating controls. The quality of a method is assessed by calculating the $\text {EC}_{20}$ value and comparing it to the known true value of the corresponding concentration-response curve.

**Fig. 2:**
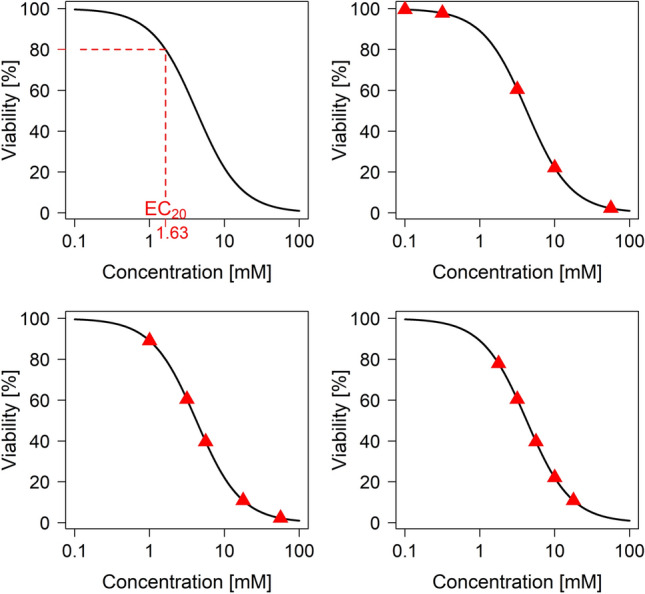
Underlying sigmoidal curve of the simulation study with true EC$_{20}$ value (top left), and selected concentrations (denoted as red triangles) for the three situations ”easy” (top right), ”medium” (bottom left) and ”difficult” (bottom right)

The shape of the true concentration-response curve is based on a real data example, which is presented in section Application to Real Data. The parameter values of the true 4pLL model are $b=1.462$, $c=0$, $d=100$, and $e=4.22$, corresponding to a decreasing curve with upper asymptote $100\%$, lower asymptote $0\%$ and inflection point 4.22, which coincides in this case with the $\text {EC}_{50}$ value. For this model, the $\text {EC}_{20}$ value is 1.63. Figure 2 (top left) visualizes the corresponding curve.

For simulating data from the true curve, we selected 5 values and the control 0 as concentrations, with 3 replicates for each concentration. Random normal noise was added to each value, with mean $\mu =0$ and standard deviation $\sigma$. The deviation of the controls was modeled by adding a shift value $\varDelta$ to the control values. The two variable parameters in this simulation study are the standard deviation $\sigma$ and the shift $\varDelta$. We set the standard deviation $\sigma$ to the values $\{2,4,8,12\}$ and the deviation $\varDelta$ of the controls from the true curve to the values $\{-10, -8, -6, -4, -2, 0, 2, 4, 6, 8, 10\}$.

We considered three situations that differ in the five concentration values with respect to the true curve. The three situations are labeled ”easy”, ”medium” and ”difficult”, see Fig. 2.

- In the ”easy” situation the concentration values cover the entire range of the curve, particularly the region around the upper asymptote. Two concentrations are in the low or no effect range, one concentration corresponds to approximately $60\%$ viability, and two concentrations are in the range of higher toxicity.
- In the ”medium” situation, only one concentration is in the low effect range, and even for this concentration the viability has already dropped by about $10\%$. Instead, two concentrations are in the middle range of the curve and two concentrations in the range of high toxicity, with only $0-10\%$ viability remaining.
- In the ”difficult” situation, neither the upper nor the lower asymptote is covered well by the concentration values. Instead, all five values are in the range of $10\%$ to $80\%$ viability.

For each combination of $\sigma$ and $\varDelta$ and for each situation, we simulated $5\,000$ datasets. We applied all four fitting methods to each of these datasets and estimated the $\text {EC}_{20}$ values. As quality measure of a model fit, the difference between the estimated $\text {EC}_{20}$ value and the true value 1.63 was calculated.

### VPA cytotoxicity study

We applied all four methods compared in the simulation study to a real dataset. The dataset is a cytotoxicity assay of the compound valproic acid (VPA), which was measured for a negative control and for 12 increasing concentrations from 0.1 mM to 56.2 mM.

Cytotoxicity with HepG2 cells was analysed using the CellTiter-Blue (CTB) assay as described in Gu et al. (2018) according to the SOP in Supplement 3A. HepG2 cells were cultivated in Dulbecco’s Modified Eagle’s Medium (DMEM) with 25 mM glucose (Albrecht et al. 2019). VPA (CAS number 99-66-1; Sigma Aldrich; product number: PHR1061-1G) was directly dissolved in the culture medium to generate the concentrations indicated in the results section so that no solvent was required.

In the experimental study, the viability was measured for seven technical replicates for each of the concentrations. To apply the four methods to datasets that resemble those from the simulation study, we chose three combinations of five concentrations analogously to the three situations ”easy”, ”medium” and ”difficult” from the simulation study. For each of the chosen concentrations we randomly sampled three out of the seven replicates.

## Results

### Literature review

In total, 2199 papers from the Archives of Toxicology (ArchTox, all issues 2016-2018), Toxicological Sciences (ToxSci, all issues 2017-2018) and Toxicology in Vitro (ToxVitro, all issues 2015-2017) were reviewed.

In Table 1, different numbers of papers and numbers of curves are listed. The first block gives the number of papers per journal, the number of papers with curves, and the number of papers that contain at least one curve fulfilling the criteria defined in section Literature Review. The second block contains the number of curves, the number of curves fulfilling the criteria, and the number of curves with indicated standard deviation. The two latter numbers differ, as for some plots it could not be detected in the respective publication whether standard deviation or standard error is shown in the plots, and in some cases with standard errors the number of replicates remains unknown.

**Table 1:** Key figures of the literature review: The table comprises the total number of papers identified in the respective years in the three journals ArchTox, ToxSci and ToxVitro, the number of papers that contain modelled dose-response curves, the number of papers where at least one curve fulfilled the criteria, the total number of modelled dose-response curves, the number of curves that fulfill the criteria, and the number of curves that fulfill the criteria and indicate whether standard deviation or standard error of the mean is indicated in the plot

	ArchTox (2016–2018)	ToxSci (2017–2018)	ToxVitro (2015–2017)
Total number of papers	810	592	797
Number of papers with curves	31	7	37
Number of papers with at least one curve fulfilling the criteria	15	6	26
Number of curves	702	65	345
Fulfilling the criteria	440	56	213
Number of curves with indicated measure of dispersion	266	56	202

The number of curves per paper is in the range 1 to 204. The median value of curves per paper fulfilling all criteria is 6 and the mean value is 17.04, with standard deviation 33.31. The three papers with the most curves fulfilling all criteria contain 204, 91, and 57 such curves, while 8 papers contain only one such curve.

**Fig. 3:**
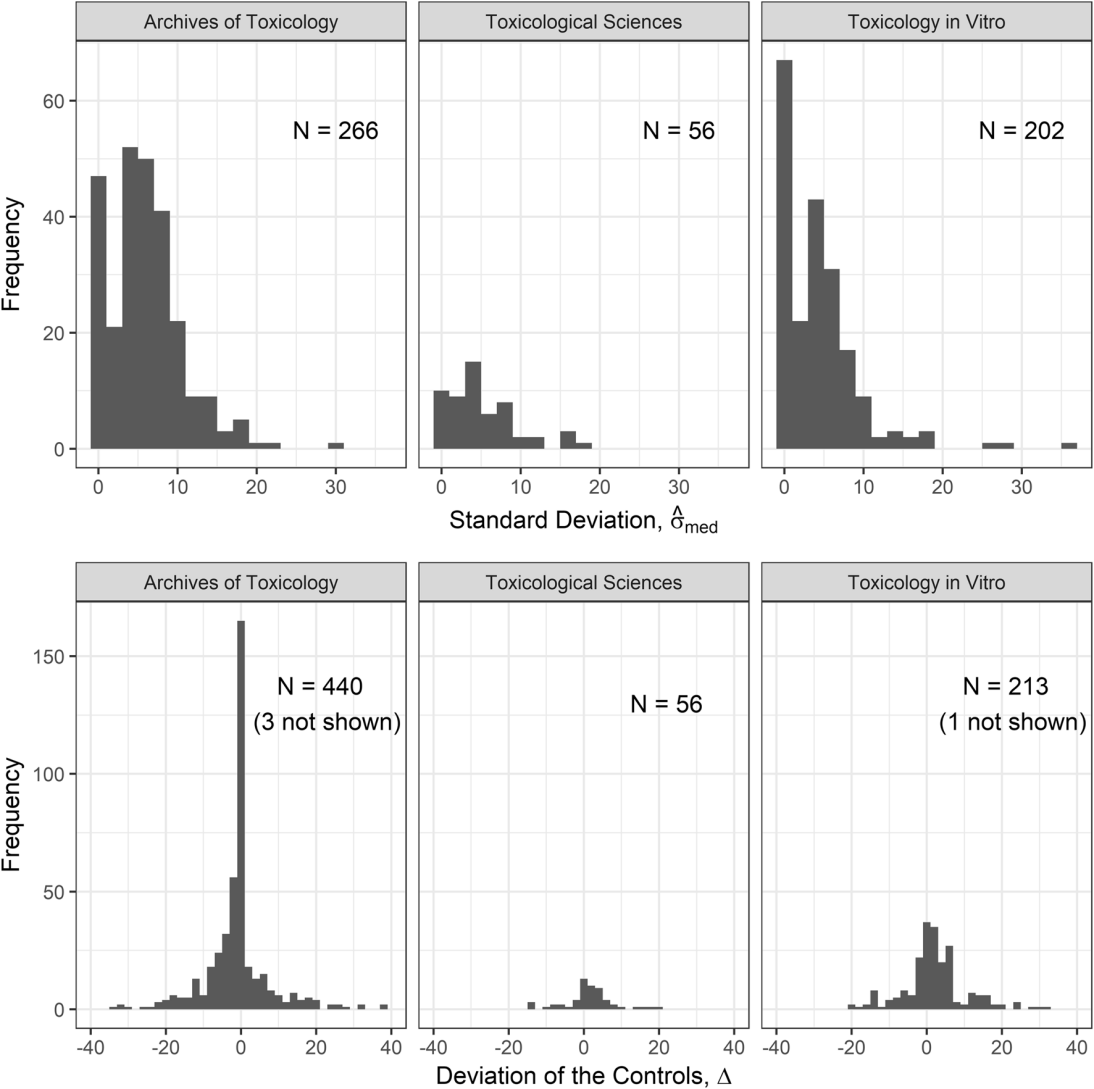
Distribution of the values of the standard deviations $\hat{\sigma }_{\text{ med }}$ and the deviations of the controls $\varDelta$ as found in ArchTox (2016–2018), ToxSci (2017–2018) and ToxVitro (2015–2017). For $\varDelta$, all curves that fulfill the criteria are considered, regardless of whether it is clearly stated whether standard deviation or standard errors are indicated. 3 values for ArchTox and 1 value for ToxVitro are larger than 40 and are not shown in this plot

Histograms for the observed values of $\varDelta$ and $\hat{\sigma }_\mathrm{med}$ illustrate the extent of deviations between the controls and the asymptote for very small concentrations (Fig. 3).

The standard deviation is in the range 0 to 10 for 85% (ArchTox), 88% (ToxSci) and 91% (ToxVitro) of the curves and in the range 0 to 20 for 99%, 100% and 99% of the curves, respectively.

The deviation of the controls is very small ($\varDelta$ in the range -2 to 2) and thus essentially negligible only for 47% (ArchTox), 38% (ToxSci) and 31% (ToxVitro) of the curves. For 80%, 88% and 79% of the curves, respectively, $\varDelta$ is in the range -10 to 10. Based on this observation, the deviation of the controls considered in the simulation study varies in that same range. Basically all deviations are between -40 and 40, only for 3 curves in ArchTox and 1 curve in ToxVitro the deviation is larger than 40.

The controls are not consistently deviating in the same direction across the three journals: For ArchTox, negative deviations with $\varDelta < -2$ occur for 34% and positive deviations with $\varDelta > 2$ for 19% of the curves. For ToxSci, negative deviations occur for 21% and positive deviations for 41% of the curves, and for ToxVitro, negative deviations occur for 23% and positive deviations for 46% of the curves.

These analyses demonstrate that deviations of the control occur frequently for concentration-response curves published in the toxicological literature.

### Simulation study

The results of the simulation study are analyzed in two different ways. In the first analysis, the proportion of estimated $\text {EC}_{20}$ values that are in an acceptable range of the true value is calculated. A factor of at most 1.3 is defined to be acceptable, i.e. the estimate of the true value $\text {EC}_{20}=1.63$ must be in the interval [1.25, 2.12].

**Fig. 4:**
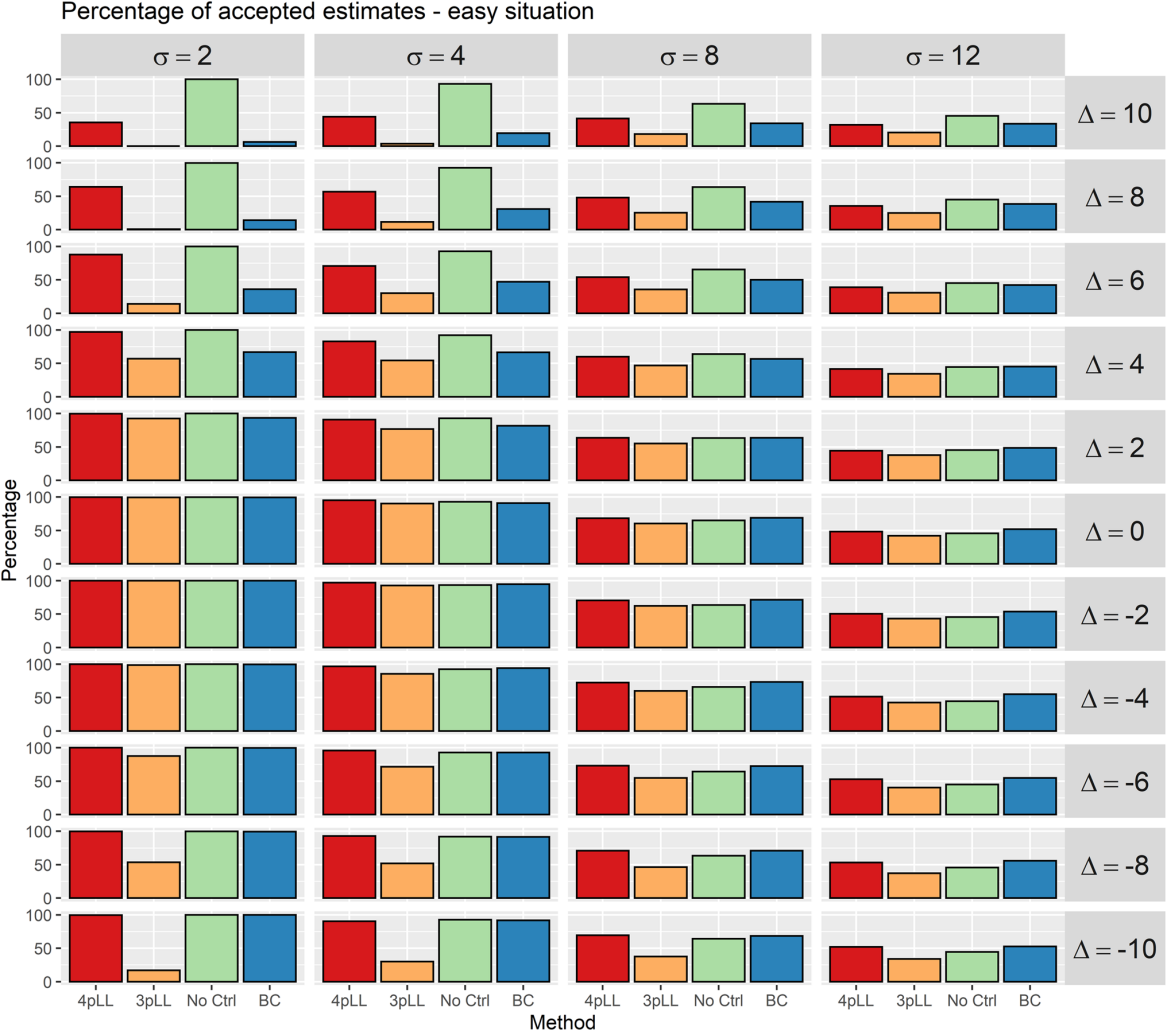
Percentages of accepted estimates for the 5000 iterations of the simulation study. In the situation shown here, the five concentrations considered cover the entire range of the curve (”easy” situation). An estimate is accepted if it takes values in the interval [1.25, 2.12]. Each cell corresponds to one combination of the standard deviation of the replicates, $\sigma$, and the deviation of the controls, $\varDelta$. In each cell, percentages for all four methods (from left to right: 4pLL, 3pLL, NoCtrl, BC) are shown. The columns represent standard deviations (increasing from left to right) and rows deviations of the controls (positive deviations on top, no deviations in the center and negative deviations in the bottom)

The proportions of accepted estimates for the ”easy” situation are shown in Fig. 4. The corresponding plots for the ”medium” and the ”difficult” situation are shown in the Supplemental Figures S1 and S2. Each cell of one plot corresponds to one combination of the parameters $\sigma$ and $\varDelta$. The columns represent from left to right increasing noise. The rows represent the deviation of the controls, with decreasing values from top to bottom and no deviation in the center.

In the ”easy” situation the methods ”4pLL” and ”No Ctrl” achieve the highest proportions. Especially for $\sigma \le 8$ and $\varDelta >6$ ”No Ctrl” outperforms also ”4pLL”. The method ”3pLL” performs clearly worse for $\sigma \ge 8$ and for large deviations ($\varDelta \le -6$ and $\varDelta \ge 4$), The method ”BC” is competitive, except for large $\varDelta$ values.

In the ”medium” situation, in general the methods ”4pLL”, ”3pLL” and ”BC” perform similar, with still about $100\%$ accepted estimates for $\sigma =2$. ”No Ctrl” often performs worse. However, for $\sigma \le 4$ and large deviations $|\varDelta |\ge 8$ ”No Ctrl” achieves clearly higher proportions.

In the ”difficult” situation, in general the results are similar to the ”medium” situation, but ”No Ctrl” only performs best for $\sigma =2$ and $|\varDelta |=10$, and now ”BC” leads to acceptable results more often for $\varDelta \le -8$, i.e. for control values with a negative deviation.

In the second analysis, we determined for each of the 5000 iterations for every parameter combination the method with the smallest absolute difference between estimated and true $\text {EC}_{20}$ value. Only iterations were considered where at least one method (and thus also the best) leads to an acceptable result.

**Fig. 5:**
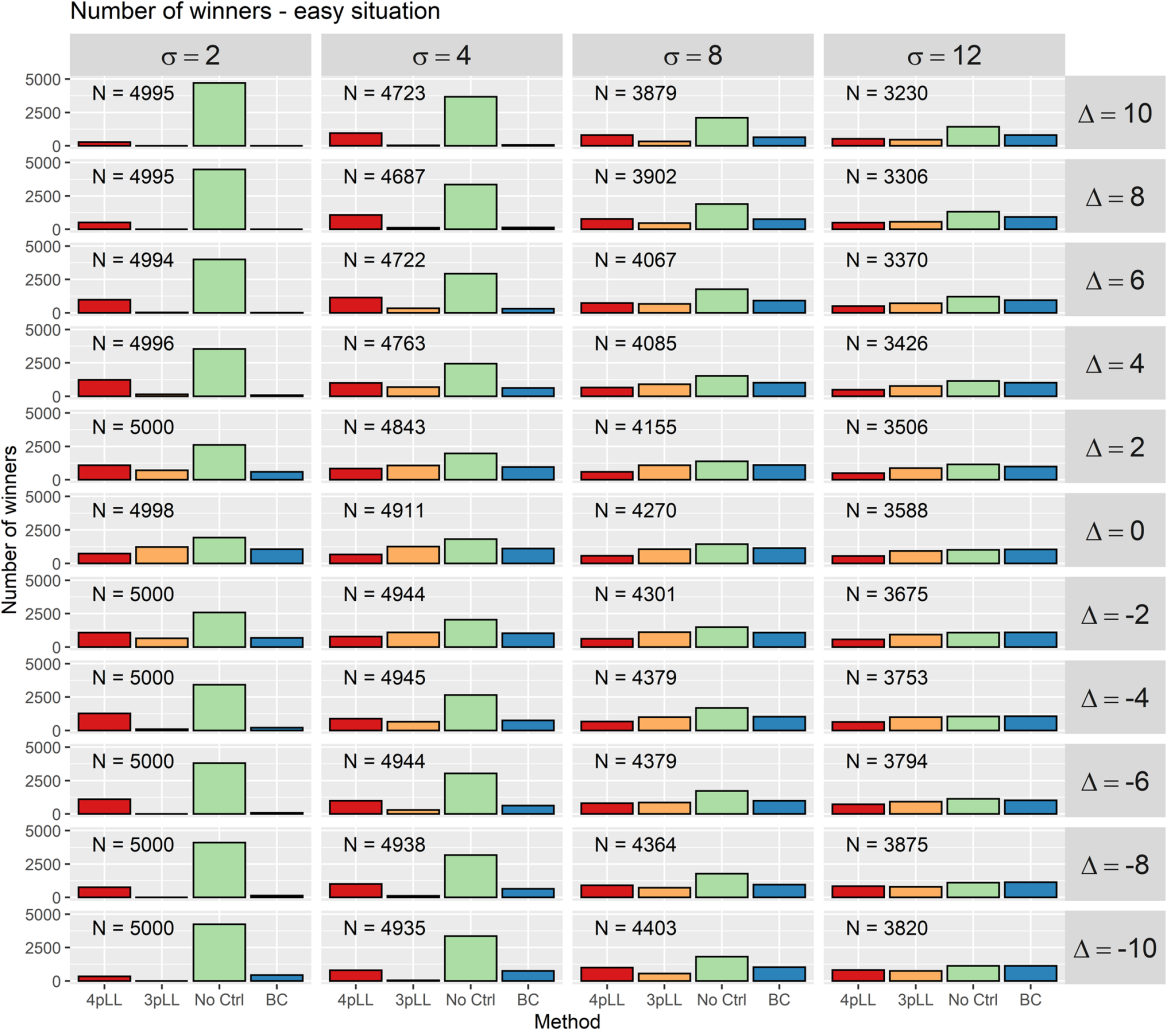
Number of times each model leads to the best estimate of the EC$_{20}$ in the 5000 iterations of the simulation study, respectively. In the situation shown here, the five concentrations considered cover the entire range of the curve (”easy” situation). Each cell corresponds to one combination of the standard deviation of the replicates $\sigma$ and the deviation of the controls $\varDelta$. In each cell, the numbers for all four methods (from left to right: 4pLL, 3pLL, NoCtrl, BC) are shown. The columns represent standard deviations (increasing from left to right) and rows deviations of the controls (positive deviations on top, no deviations in the center and negative deviations in the bottom). *N* denotes the number of iterations in which at least one method lead to an estimate in the interval [1.25, 2.12] (”acceptable result”)

For the ”easy” situation, for every combination of the parameters $\sigma$ and $\varDelta$, the frequencies how often the methods perform best, respectively, are shown in Fig. 5. For the ”medium” and ”difficult” situation, corresponding plots are shown in Supplemental Figures S3 and S4. The number in each cell gives the number of iterations in which at least one method lead to an acceptable estimate of the $\text {EC}_{20}$ value.

The ”easy” and the ”medium” situation lead overall to very similar conclusions. In almost all cases, ”No Ctrl” is by far the most frequent winner, especially for small $\sigma$ and large absolute $\varDelta$ values. From the other methods, ”4pLL” often is the best one, while ”3pLL” and ”BC” fail more often.

For moderate $\varDelta$, ”No Ctrl” still is the best method most frequently. This is even true for $\sigma =2$ and $\varDelta =0$ in the ”easy” situation, where controls are expected to help. However, the other methods also lead to the smallest error in estimating the $\text {EC}_{20}$ considerably often.

In the ”difficult” situation, the results for $\sigma \ge 8$ look similar across all values of $\varDelta$. ”No Ctrl” rarely is the best method, whereas all other three methods win similarly often. ”BC” slightly dominates for negative values of $\varDelta$. The more extreme the deviation is in this direction, the better is the method ”BC”.

For $\sigma =2$ and $\sigma =4$, the results are different. For large $\varDelta$ ”No Ctrl” is most frequently the best method, in a medium range of $\varDelta$ often ”4pLL” is the winner, while ”No Ctrl” performs poorly, and for negative $\varDelta$, ”BC” is the best method.

In summary, the main results are:

- In the ”easy” situation ”4pLL” and ”No Ctrl” are competitive, but for large deviations of the controls and small standard deviation of the replicates ”No Ctrl” is clearly better.
- In the ”medium” situation, we have to distinguish between numbers of acceptable estimates and winners. ”No Ctrl” is often the winner, but it leads to acceptable results less frequently, except for large deviations and small standard deviations.
- In the ”difficult” situation, ”4pLL” is competitive, but for very large positive deviations ”No Ctrl” performs better and for large negative deviations ”BC” performs better.

In addition to these analyses for the EC$_{20}$, we conducted the same analyses for the EC$_{50}$. The true value of the EC$_{50}$ for the underlying curve (Fig. 2) is 4.22. Again, plots summarizing the proportion of acceptable results (Supplemental Figures S5, S6 and S7) and plots counting the number of winners (Supplemental Figures S8, S9 and S10) are created. Since estimation of the EC$_{50}$ is affected less by deviation of the controls, a narrower acceptable range around the true EC$_{50}$ is considered: A factor of at most 1.1 is defined to be acceptable, i.e. the estimate of the true value EC$_{50} = 4.22$ must be in the interval [3.84, 4.64].

Regarding the percentage of accepted estimates, the results are very similar to those of the EC$_{20}$ in all three situations with equally slightly less accepted estimates. For $\sigma \ge 8$ or $|\varDelta | \le 4$, the methods ”3pLL”, ”4pLL” and ”BC” perform similarly. A difference between the three scenarios can be observed for ”No Ctrl”, with fewer acceptable results for medium and difficult situation.

For the number of winners, an increase in the number of times that ”3pLL” leads to the best result is noticeable. In the case of $|\varDelta |\le 2$, ”3pLL” is the best method most often, while for larger deviations ”No Ctrl” and ”BC” lead to the best result most often, in the ”easy” situation also ”4pLL” is competitive.

One point of criticism regarding counting the number of winners is that no differences between the performances of the methods are shown: It may happen that one method only very slightly dominates another method in the majority of cases, but this other method is far better than the first method in the remaining cases. In such a scenario we would prefer the second method over the first one, but the number of winners suggests otherwise. Therefore, this analyses should be interpreted with caution and algorithmic recommendations should mainly follow the analysis considering the percentage of acceptable results. The recommendations remain unchanged by the analysis of the EC$_{50}$.

### Application to real data

**Fig. 6:**
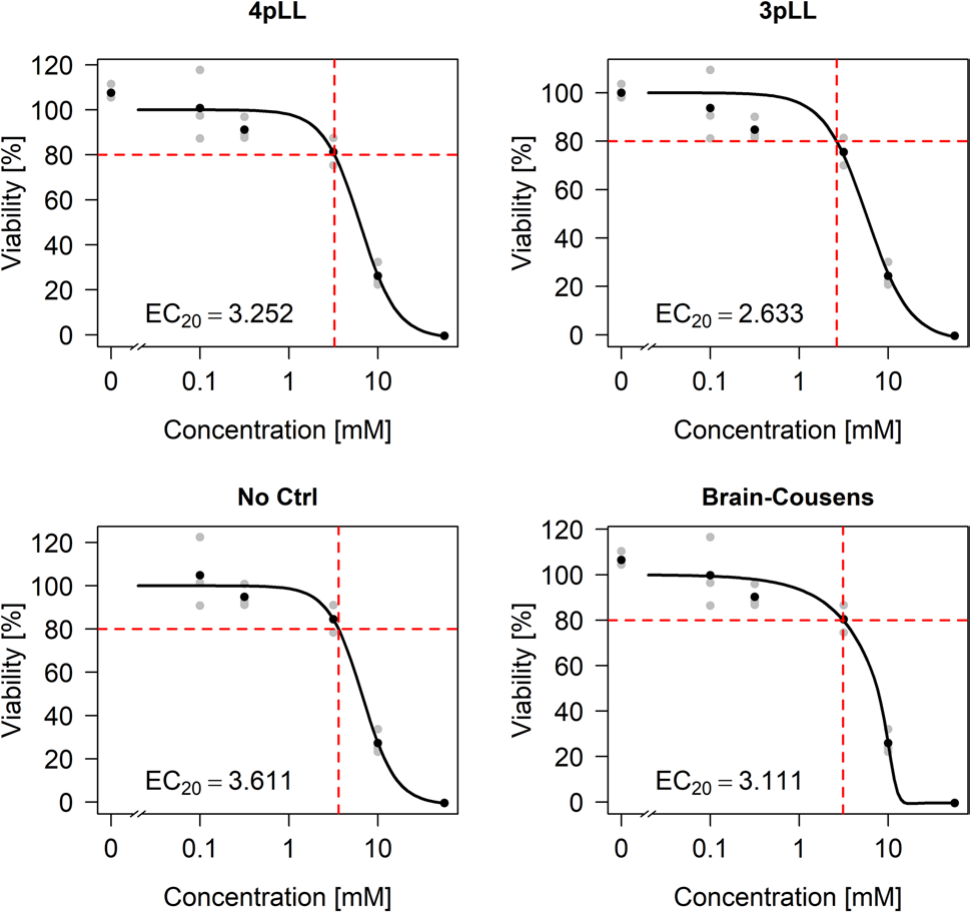
Application of the four methods ”4pLL”, ”3pLL”, ”NoCtrl” and ”BC” to a part of the VPA dataset according to the ”easy” situation from the simulation study

The four procedures described in section Handling deviating controls were applied to the VPA dataset, resembling the ”easy” situation (Fig. 6). The plots for the ”medium” and the ”difficult” situation are shown in Figures S11 and S12 in the supplement. For the ”easy” situation, we observe a positive deviation of the controls with $\varDelta \approx 12$. For the other two situations, reliable estimation of $\varDelta$ directly from the reduced dataset is not possible. In the ”easy” situation, the standard deviations lie approximately in the interval [2,11.5] with a median close to 5. In the ”medium” and ”difficult” situation the standard deviations are smaller, with median values around 2.1 and 4.3, respectively.

The estimated EC$_{20}$ values for the three situations and for the four modeling methods are presented in Table 2. In all three situations, the method ”No Ctrl” leads to the largest estimates for the EC$_{20}$. For the ”easy” and the ”medium” situation, this is followed by the method ”4pLL”, while ”3pLL” leads to the smallest estimates in all situations. The estimates for ”No Ctrl” and ”3pLL” differ by a factor of 1.3 in the ”easy” situation, by a factor of 2.3 in the ”medium” situation and by a factor of 1.7 in the ”difficult” situation. The estimation method thus heavily influences the resulting estimate of the EC$_{20}$, even in the ”easy” situation.

**Table 2:** EC$_{20}$ values for all four methods applied to parts of the VPA dataset according to the ”easy”, the ”medium” and the ”difficult” situation from the simulation study

	4pLL	3pLL	No Ctrl	BC
Easy	3.25	2.63	3.61	3.11
Medium	2.45	1.77	3.99	1.96
Difficult	1.87	1.85	3.23	1.91

The curve modelled with ”BC” is not necessarily monotonously decreasing. For example, in the ”medium” and the ”difficult” situation, the modelled curve takes its lowest value between the two highest concentrations and increases again. The value of the right asymptote in the ”middle” situation is 209 and even 945 in the ”difficult” situation, which is biologically not meaningful and which illustrates one of the problems when modelling a Brain-Cousens curve in a situation where the controls are not negatively deviating.

## Discussion and conclusions

Fitting curves to estimate the relationship between concentrations of a compound and the viability of cells is a frequent task in the analysis of toxicological and biological assays. Control values corresponding to concentration 0 are often used to normalize the estimated response values before or after curve fitting. However, from experience and based on our review of the recent toxicological literature it becomes obvious that often response values of the control concentration do not fit well to asymptotes of the fitted curves in the low-concentration range. Such deviating controls must be accounted for in the statistical analysis. We focused on the quality of estimates of effective concentrations derived from the fitted curves.

The simulation study helps to understand in which theoretical situations which method is best. However, since the true function is not known in practical data situations, only features of the observed data can be used to make decisions on the analysis strategy. The standard deviation of the replicates can be estimated from the data, but the deviation of the controls cannot be estimated in general, especially without concentrations in the no-effect range. In the ”easy” situation, there are two no-effect concentrations that help to determine the upper asymptote and to estimate the deviation. In the ”medium” and ”difficult” situation this is only possible for small standard deviations. In the ”difficult” situation there is still a clear decrease in the response for the two lowest concentrations. If the observed response value for the control is only slightly larger than the observed values for the lowest concentration, this can be interpreted as negatively deviating controls.

Our comparisons of different approaches on simulated and real data lead to clear recommendations how to address the challenge of deviating controls. In general, for true sigmoidal relationships as expected in many real world scenarios, the popular ”4pLL” approach works well. In case of a high-quality fit of the asymptote for low concentrations, low variances of the replicate measurements, and deviating controls, the approach ”No Ctrl” (omit control values) clearly leads to better results. In case of missing values both in the low-concentration and in the high-concentration range, ”4pLL” or ”BC” lead to the best results and should both be considered. As biologically meaningless results may occur when modeling a BC curve with positively deviating controls, this method should only be used for negative deviations and should always be subjected to a plausibility check.

In general we strongly discourage the use of the ”3pLL” method as it performs clearly worse in terms of percentage of accepted estimates in comparison to the other methods. Only for estimation of the EC$_{50}$, where the value of the upper asymptote has less impact than for estimating the EC$_{20}$, ”3pLL” leads to the most accurate estimate most often especially in those situations where all methods perform similarly regarding accepted estimates.

**Fig. 7:**
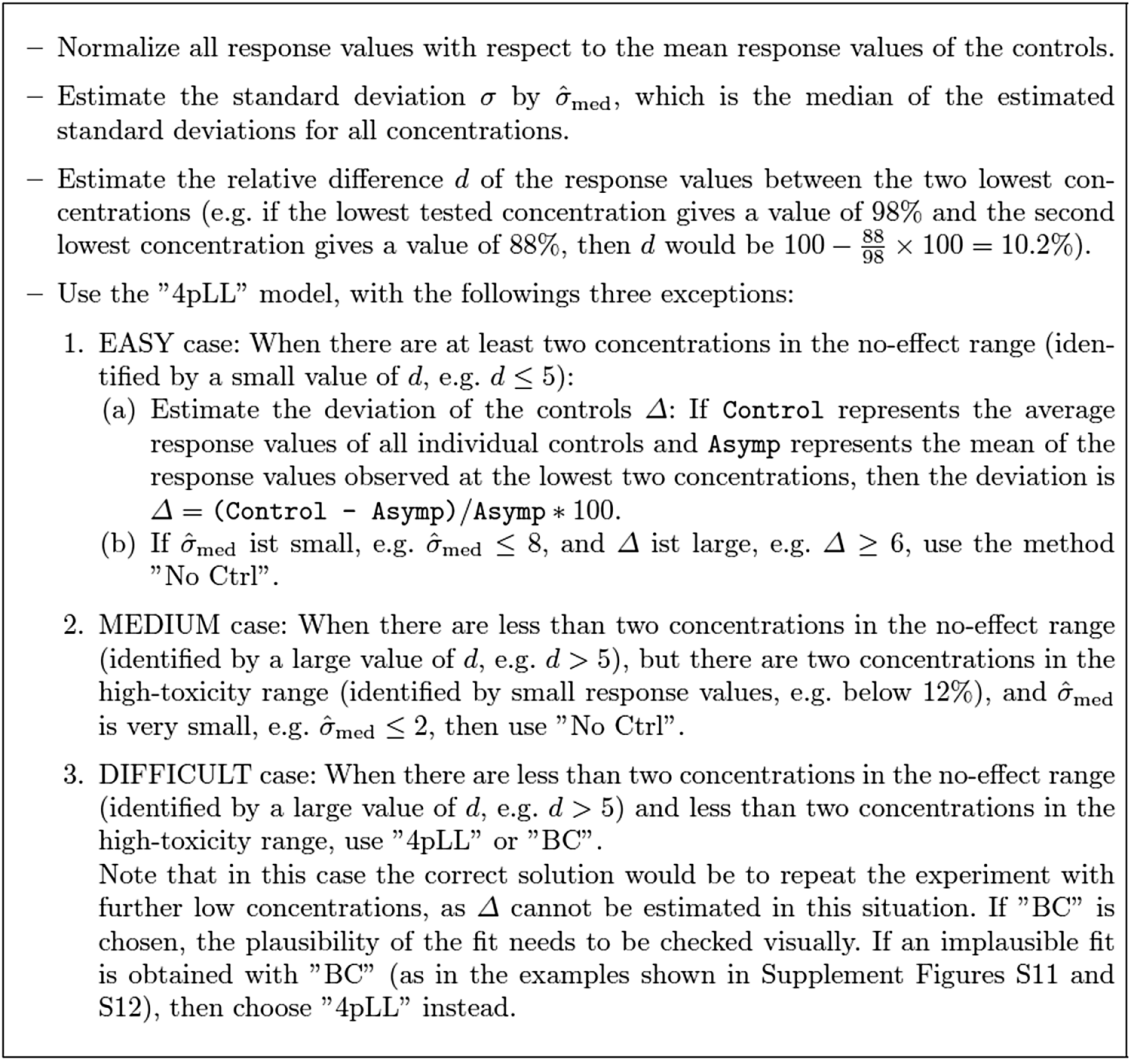
Recommended algorithm for fitting concentration-response curves to toxicological data from viability or cell function assays, considering potential deviations for the negative control. The algorithm is specifically aimed at the situation of monotonously decreasing concentration-response-curves

Note that we specifically investigated the situation of monotonously decreasing concentration-response-curves. For this scenario, as practical guideline for the analysis of a real dataset, we propose the procedure as presented in Fig. 7.

Based on these recommendations, for the application to the VPA dataset, in the ”easy” situation the method ”No Ctrl” should yield the best estimate of the EC$_{20}$, which is in this case 3.61mM. Looking at the full dataset, the controls seem to be positively deviating such that ”No Ctrl” indeed seems to be the most plausible and suitable method for this case.

## Electronic supplementary material

Below is the link to the electronic supplementary material.

Supplementary material 1 (PDF 155 kb)
