# Supplementary material for: Handling deviating control values in concentration-response curves
Source: Arch Toxicol. 2020 Sep 23;94(11):3787–98. doi: 10.1007/s00204-020-02913-0 (PMC7603474; doi:10.1007/s00204-020-02913-0)
Supplement: Supplementary file 1 — Supplementary material 1 (PDF 155 kb) [file 204_2020_2913_MOESM1_ESM.pdf]

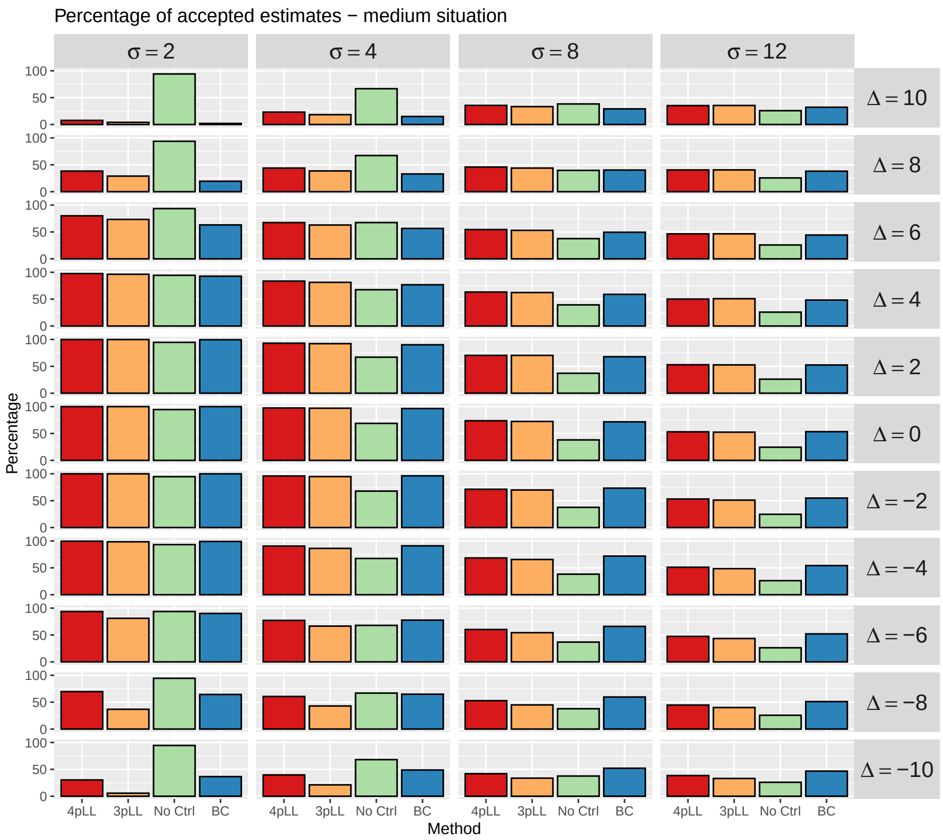

Suppl. Fig. 1 – Percentage of accepted estimates for the EC_{20} in the medium situation. Each cell corresponds to one combination of σ and Δ. The columns represent standard deviations (increasing from left to right) and rows deviation of the controls (positive deviations on top, no deviations in the center and negative deviations in the bottom).

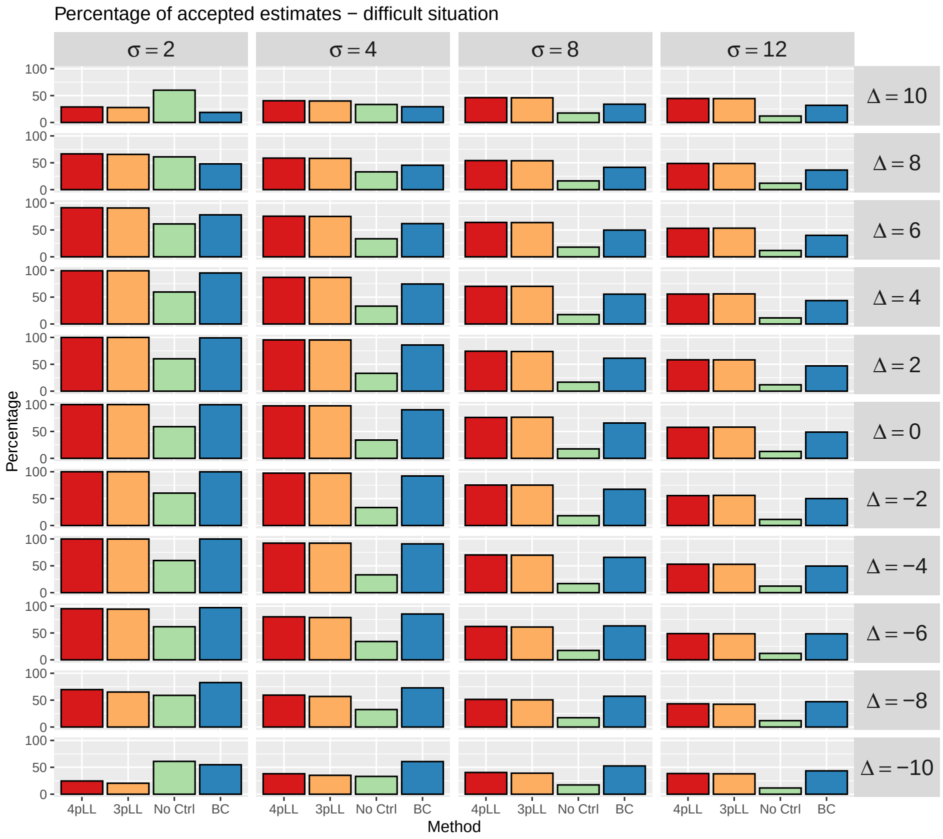

Suppl. Fig. 2 – Percentage of accepted estimates for the EC_{20} in the difficult situation. Each cell corresponds to one combination of σ and Δ. The columns represent standard deviations (increasing from left to right) and rows deviation of the controls (positive deviations on top, no deviations in the center and negative deviations in the bottom).

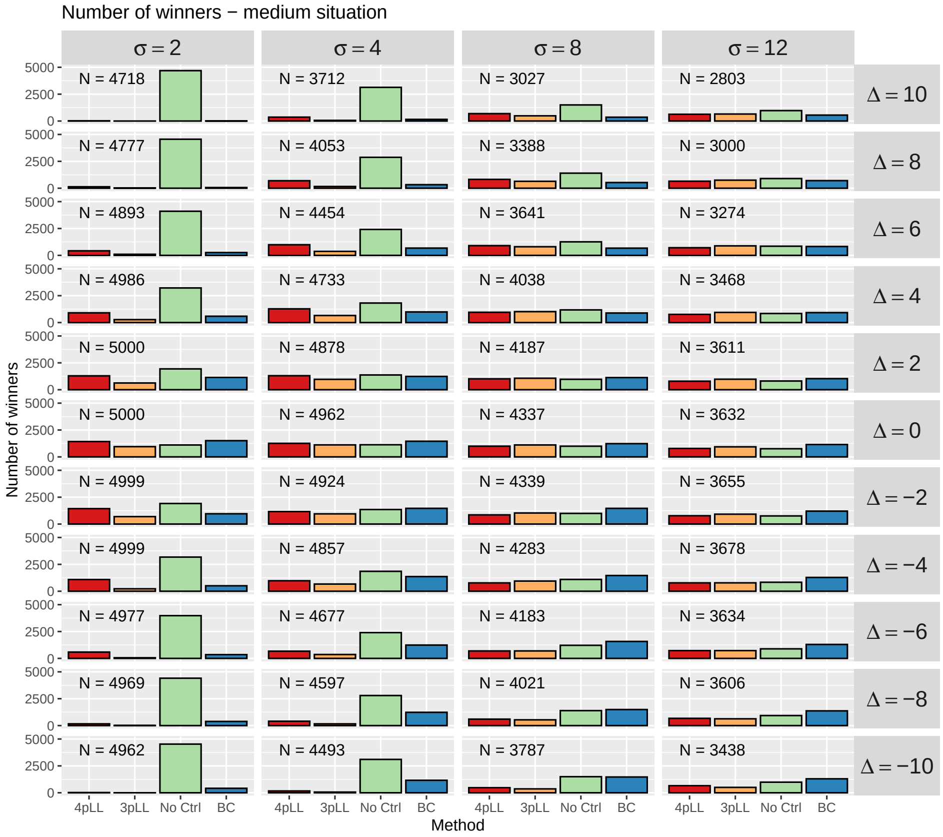

Suppl. Fig. 3 – Number of winners for the EC_{20} for the medium situation. Each cell corresponds to one combination of σ and Δ. The columns represent standard deviations (increasing from left to right) and rows deviation of the controls (positive deviations on top, no deviations in the center and negative deviations in the bottom). N denotes the number of iterations in which at least one method lead to an acceptable result.

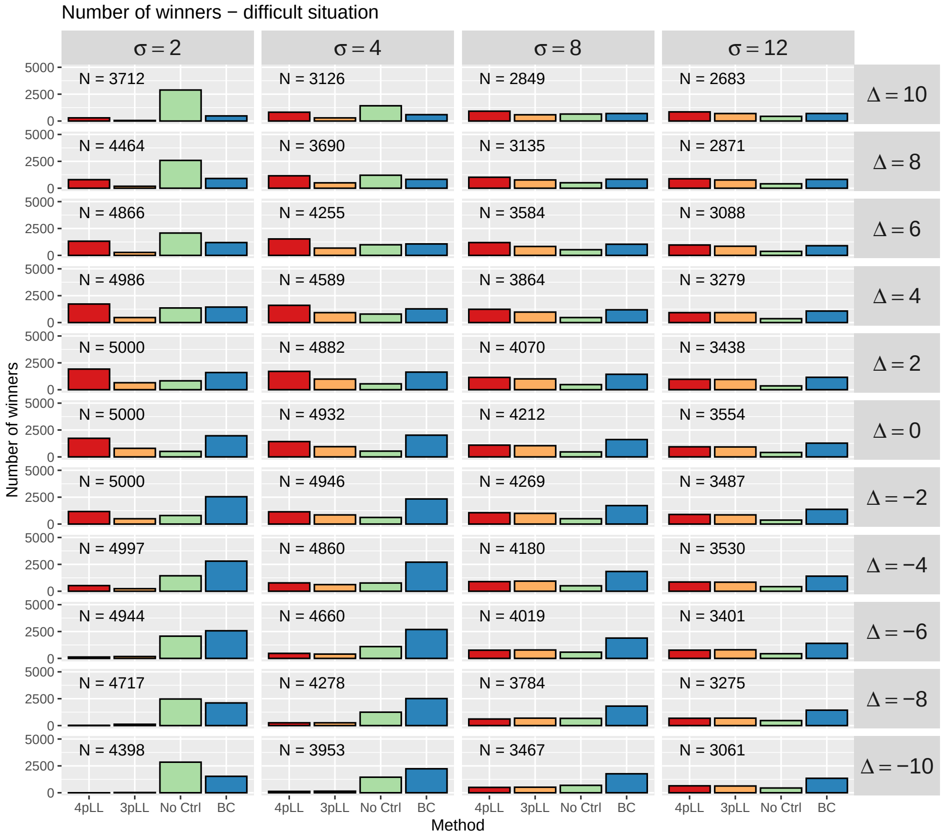

Suppl. Fig. 4 – Number of winners for the EC_{20} for the difficult situation. Each cell corresponds to one combination of σ and Δ. The columns represent standard deviations (increasing from left to right) and rows deviation of the controls (positive deviations on top, no deviations in the center and negative deviations in the bottom). N denotes the number of iterations in which at least one method lead to an acceptable result.

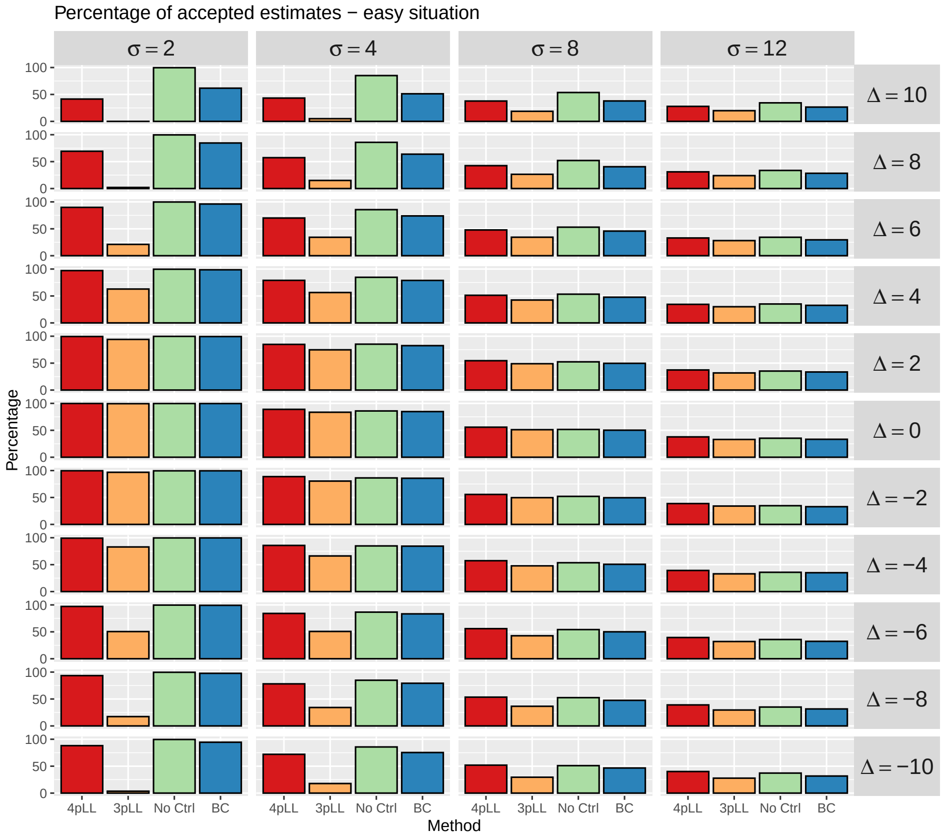

Suppl. Fig. 5 – Percentage of accepted estimates for the EC_{50} in the easy situation. In contrast to results for the EC_{20}, the range of acceptable results is narrower with a factor of 1.1, i.e. an estimate is acceptable if it lies in the interval $[3.84, 4.64]$ around the true underlying EC_{50} of 4.22. Each cell corresponds to one combination of σ and Δ. The columns represent standard deviations (increasing from left to right) and rows deviation of the controls (positive deviations on top, no deviations in the center and negative deviations in the bottom).

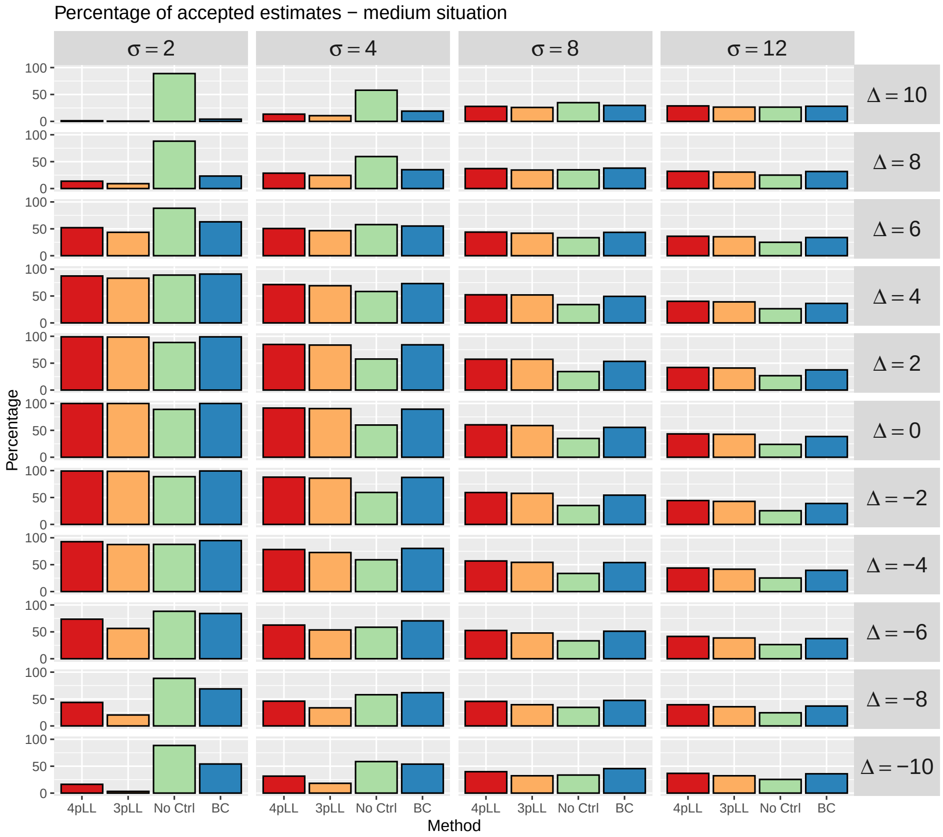

Suppl. Fig. 6 – Percentage of accepted estimates for the EC_{50} in the medium situation. In contrast to results for the EC_{20}, the range of acceptable results is narrower with a factor of 1.1, i.e. an estimate is acceptable if it lies in the interval $[3.84, 4.64]$ around the true underlying EC_{50} of 4.22. Each cell corresponds to one combination of σ and Δ. The columns represent standard deviations (increasing from left to right) and rows deviation of the controls (positive deviations on top, no deviations in the center and negative deviations in the bottom).

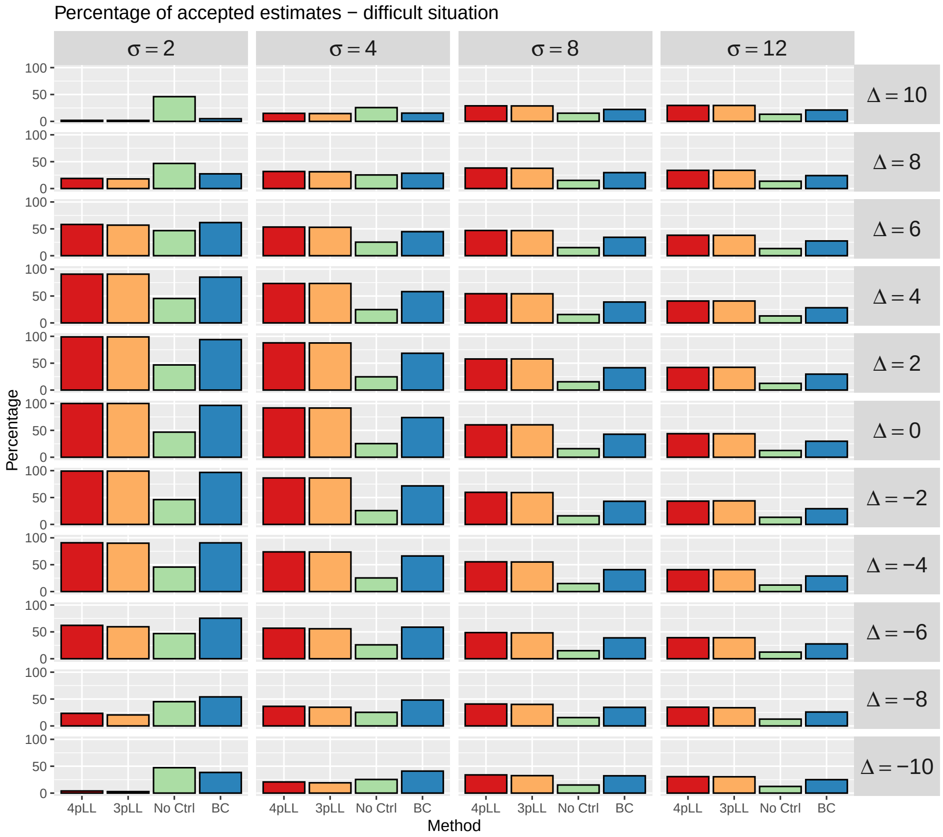

Suppl. Fig. 7 – Percentage of accepted estimates for the EC_{50} in the difficult situation. In contrast to results for the EC_{20}, the range of acceptable results is narrower with a factor of 1.1, i.e. an estimate is acceptable if it lies in the interval $[3.84, 4.64]$ around the true underlying EC_{50} of 4.22. Each cell corresponds to one combination of σ and Δ. The columns represent standard deviations (increasing from left to right) and rows deviation of the controls (positive deviations on top, no deviations in the center and negative deviations in the bottom).

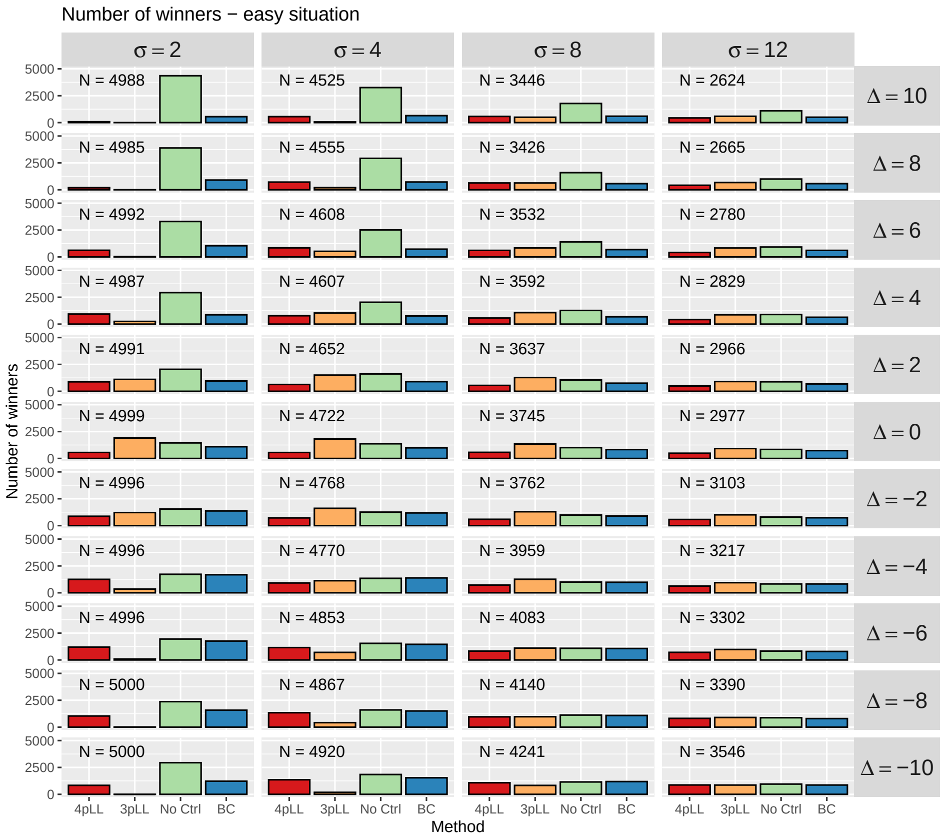

Suppl. Fig. 8 – Number of winners for the EC_{50} in the easy situation. Each cell corresponds to one combination of σ and Δ. The columns represent standard deviations (increasing from left to right) and rows deviation of the controls (positive deviations on top, no deviations in the center and negative deviations in the bottom). N denotes the number of iterations in which at least one method lead to an acceptable result, where an acceptable result is attained if the estimate lies not further away from the true underlying EC_{50} than by a factor of 1.1.

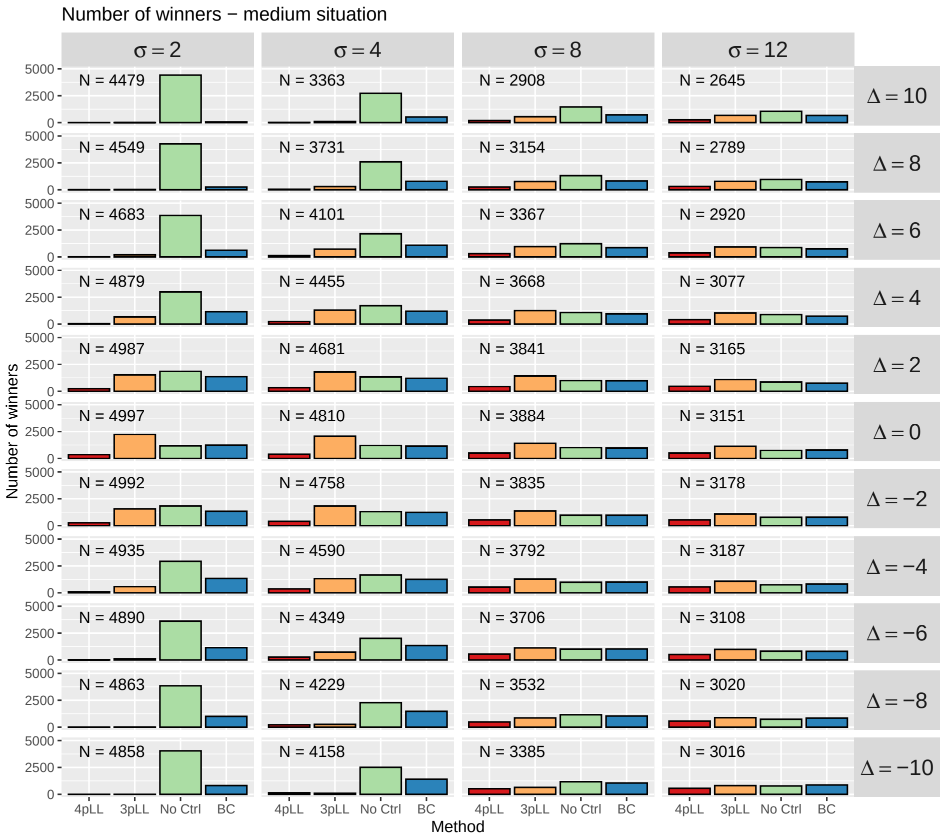

Suppl. Fig. 9 – Number of winners for the EC_{50} in the medium situation. Each cell corresponds to one combination of σ and Δ. The columns represent standard deviations (increasing from left to right) and rows deviation of the controls (positive deviations on top, no deviations in the center and negative deviations in the bottom). N denotes the number of iterations in which at least one method lead to an acceptable result, where an acceptable result is attained if the estimate lies not further away from the true underlying EC_{50} than by a factor of 1.1.

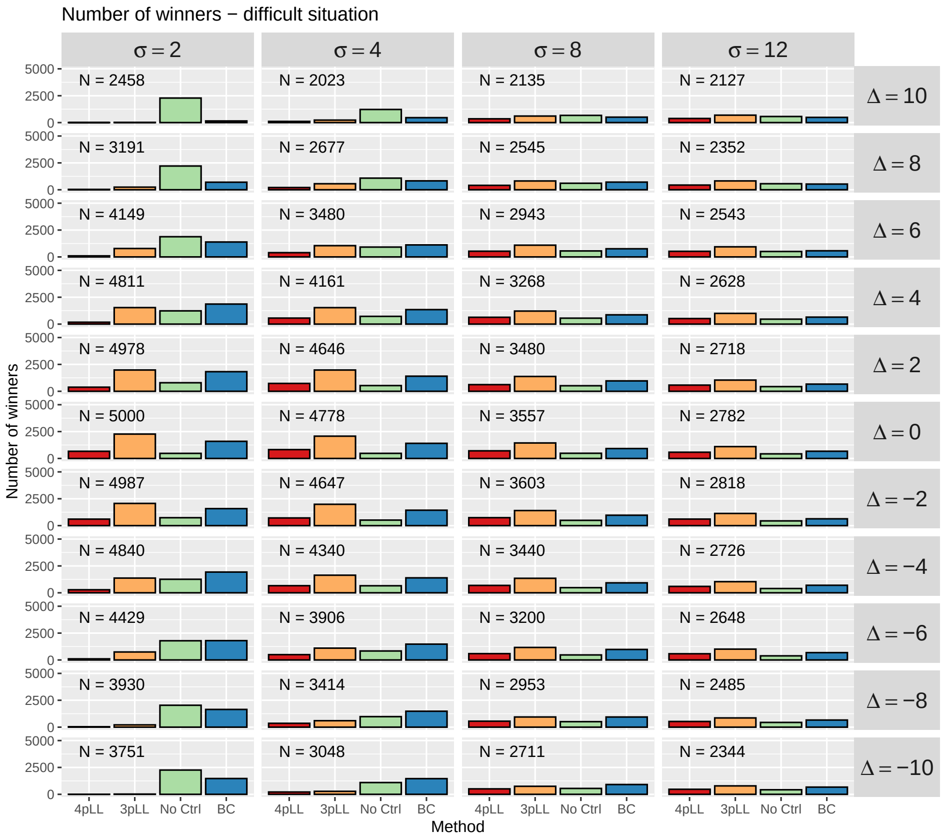

Suppl. Fig. 10 – Number of winners for the EC_{50} in the difficult situation. Each cell corresponds to one combination of σ and Δ. The columns represent standard deviations (increasing from left to right) and rows deviation of the controls (positive deviations on top, no deviations in the center and negative deviations in the bottom). N denotes the number of iterations in which at least one method lead to an acceptable result, where an acceptable result is attained if the estimate lies not further away from the true underlying EC_{50} than by a factor of 1.1.

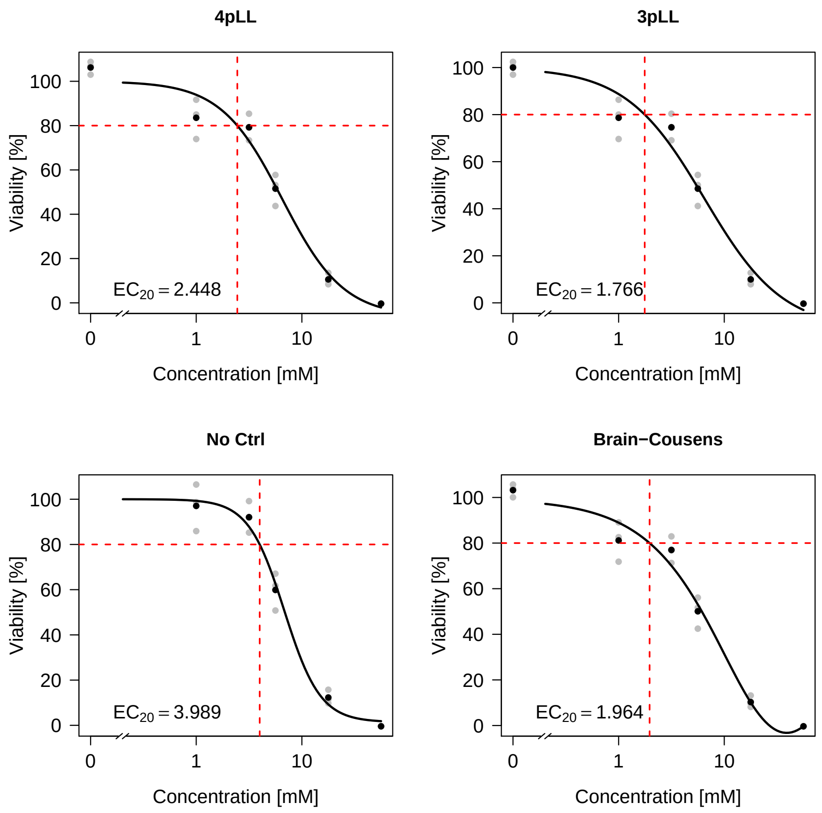

Suppl. Fig. 11 – Application of all 4 methods to a part of the VPA dataset that resembles the "medium" situation from the simulation study.

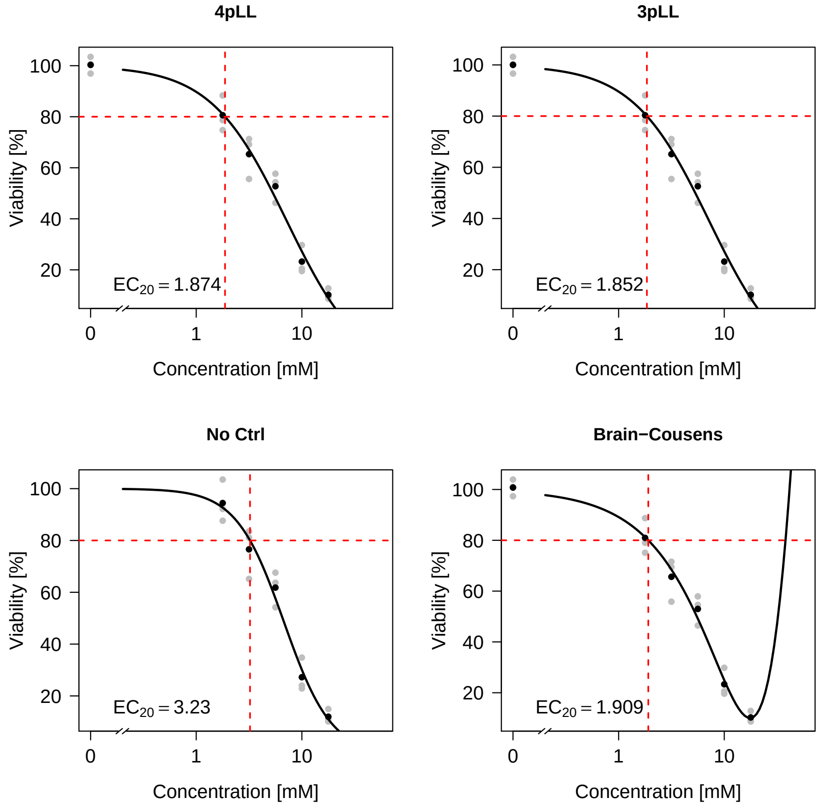

Suppl. Fig. 12 – Application of all 4 methods to a part of the VPA dataset that resembles the "difficult" situation from the simulation study.
